# Peptides From Adzuki Bean and Soybean Improved Insulin‐AKT Signaling‐Related Pathways in Healthy and Insulin‐Resistant States in Human Liver Cells

**DOI:** 10.1002/mnfr.70285

**Published:** 2025-10-09

**Authors:** Shu Hang Kwan, Elvira Gonzalez de Mejia

**Affiliations:** ^1^ Division of Nutritional Sciences University of Illinois at Urbana‐Champaign Champaign Illinois USA; ^2^ Department of Food Science and Human Nutrition University of Illinois at Urbana‐Champaign Champaign Illinois USA

**Keywords:** adzuki bean (*Vigna angularis)* β‐vignin, dipeptidyl peptidase IV inhibition, glucose uptake, insulin signaling, soybean β‐conglycinin

## Abstract

The objectives were to determine the effect and mechanism of functional peptides identified from digested adzuki bean β‐vignin and soybean β‐conglycinin on insulin‐AKT signaling and hepatic glucose uptake markers and to investigate the roles of these peptides in modulating the insulin‐AKT signaling pathway in human liver cells in healthy and insulin‐resistant states. Methods and Results: Adzuki bean β‐vignin and soybean β‐conglycinin proteins were isolated and digested using simulated gastrointestinal digestion. Peptide sequences (VP, PM, FNE, LLS, and IPA), in silico analysis and in vitro (cell‐free and HepG2 cell‐based) systems confirmed their safety and dipeptidyl peptidase IV (DPP IV) inhibitory capacity. IPA and VP showed high intestinal absorption and low toxicity, inhibited DPP IV (IPA IC_50_, 7.86 µM; VP IC_50_, 9.58 µM). Microarray results showed that VP (9.58 µM) stimulated the insulin signaling pathway in the healthy state. In healthy and insulin‐resistant states, VP (9.58 µM) and IPA (7.86 µM) significantly increased (p < 0.05) protein expression of IRS‐1, Akt‐1, and Glut 2, suggesting their potential in modulating insulin signaling and hepatic glucose uptake. Peptides exhibited antidiabetic properties by stimulating insulin signaling. These in vitro findings support further investigation into their application in functional food ingredients targeting glucose metabolism.

## Graphical abstract

This study investigated the antidiabetic potential of adzuki bean β‐vignin and soybean β‐conglycinin peptides in human liver cells in healthy and insulin‐resistant states. Peptide Val‐Pro was found to inhibit dipeptidyl peptidase IV and stimulate the insulin signaling pathway, suggesting the potential for developing healthier adzuki bean‐based functional food ingredients.

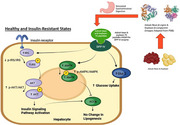

**Abbreviations**
ACCα: acetyl‐CoA carboxylase α
Akt‐1: AKT serine/threonine kinase 1
Akt1S1: AKT1 substrate 1
AMPK1/2: AMP‐activated protein kinase catalytic subunit alpha‐1/2
DLDV: Asp‐Leu‐Asp‐Val
DPP IV: dipeptidyl peptidase IV
FDA: US Food and Drug Administration
FNE: Phe‐Asn‐Glu
GAPDH: glyceraldehyde‐3‐phosphate dehydrogenase
Glut 2: glucose transporter 2
IC: inhibitory concentration
IPA: Ile‐Pro‐Ala
IR: insulin receptor
IRS‐1: insulin receptor substrate 1
LLS: Leu‐Leu‐Ser
mTOR: mechanistic target of rapamycin
PM: Pro‐Met
PP2A‐α: protein phosphatase 2 alpha
Shc: SHC‐transforming protein 1
T2D: type 2 diabetes
VP: Val‐Pro

## 1 Introduction

The global prevalence of diabetes is projected to increase by 45% by 2050 [1]. In 2022, the United States spent $413 billion (∼25% of the healthcare government spending) on medical care for people diagnosed with diabetes [2]. As such, many studies have focused on developing ways to prevent and manage type 2 diabetes (T2D). Insulin resistance is one of the most frequently studied areas. Insulin resistance is the state where insulin stimulation is impaired in the insulin‐targeting tissues, and it plays an important role in T2D development [3, 4, 5, 6, 7]. Insulin resistance increases hepatic de novo lipogenesis and contributes to the development of a fatty liver, which in turn aggravates hepatic insulin resistance [8]. Thus, it is important to develop a means that helps manage insulin resistance and thus lowers the risk of T2D.

Substituting animal protein with plant protein lowered the T2D risk and improved glycemic control in individuals with T2D [9, 10]. Legume consumption is recommended due to their high nutritional value and potential health benefits [11, 12, 13]. A human randomized controlled trial found that increased legume consumption may prevent T2D [10].

Adzuki bean (AB, *Vigna angularis*), with a glycemic index of 26, is one of the legumes that exhibits antidiabetic properties and is used in Asian cultures for T2D management [14, 15, 16, 17, 18]. Supplementing 150 mg AB flour/kg diet/day to a high‐fat diet for 12 weeks significantly decreased fasting blood glucose, fasting serum insulin, the HOMA‐IR index, and blood glucose during an oral glucose tolerance test [14]. Another study revealed similar improvements in T2D‐related outcomes in streptozotocin‐induced diabetic mice after feeding them a diet containing 30% cooked AB flour for 8 weeks [19]. An in vitro study found that digesting AB β‐vignin (AB7S) resulted in peptides with DPP IV inhibitory capacity, and treating human liver cells with 1 mg digested AB7S/mL significantly increased hepatic glucose uptake in both healthy and insulin‐resistant states [20]. Soybean (*Glycine max*) also has the potential to manage T2D outcomes. Soybean β‐conglycinin (S7S) consumption improved diabetic outcomes in rats [21]. A meta‐analysis of 15 human cohort studies reported a negative association between soy protein consumption and the risk of T2D [22].

Bioactive peptides are used as functional ingredients in food formulation because of their high purity, reproducibility, and scalability, which might not be provided by peptide‐rich digests or extracts [23, 24]. Bioactive peptides are peptides with fewer than forty amino acids that possess a bioactive function in cells [25, 26]. These peptides are found in the structure of parent proteins and are activated by protein cleavage [27]. Some of these peptides are produced in the body while others are delivered as foods, dietary supplements, and medications [28]. These peptides were found to have anti‐hypertensive, anti‐microbial, antioxidative, anti‐obesity, and antidiabetic potential [29, 30, 31, 32, 33]. In this regard, peptides can be used to manage diseases. Therapeutic peptides are one of the applications that are used to treat metabolic diseases, cardiovascular diseases, respiratory diseases, and pain, and the US Food and Drug Administration (FDA) oversees and provides recommendations to industry for therapeutic peptide development [23, 34]. The FDA approved 239 therapeutic peptides and proteins [35]. In 2023 alone, the FDA approved nine therapeutic peptides, which accounted for 16% of all approved drugs in 2023 [36]. Therefore, it is possible that functional peptides from digested AB7S and S7S could play a beneficial role in T2D management, and it is essential to understand the underlying mechanism of these peptides in modulating the T2D‐related metabolism.

Although animal and human studies reported the antidiabetic benefits provided by AB consumption, the roles AB functional peptides play in the underlying mechanisms of T2D‐related metabolism remain uncertain, as reported by Kwan and Gonzalez de Mejia and Harahap et al. [17, 37]. Although studies found that legumes could upregulate the insulin‐Akt signaling pathway [38], there is a need to study the relationship of DPP IV inhibition and the activation of the insulin‐Akt pathway for adzuki bean. Therefore, the objective of this study was to determine the effect and mechanism of pure synthetic functional peptides from digested AB7S and S7S on the insulin‐AKT signaling pathway in a liver monoculture model under both healthy and insulin‐resistant states. We hypothesized that treatments with pure functional peptides from digested AB7S and S7S would modulate the insulin‐Akt signaling and glucose uptake pathways in liver cells, enhancing their insulin signaling and glucose uptake in both healthy and insulin‐resistant states.

## 2 Experimental Section

### 2.1 Materials

The peptides we have sequenced [20] [Val‐Pro (VP), Pro‐Met (PM), Phe‐Asn‐Glu (FNE), Leu‐Leu‐Ser (LLS), and Ile‐Pro‐Ala (IPA), with >98% purity] were purchased from CPC Scientific (Rocklin, CA, USA). The human HepG2 (HB‐8065) cell line was purchased from the American Type Culture Collection (ATCC, Manassas, VA, USA). Primary antibodies for human insulin receptor substrate 1 (IgG_2a_ κ IRS‐1), AKT serine/threonine kinase 1 (IgG_1_ κ Akt‐1), acetyl‐CoA carboxylase α (IgG_2b_ κ ACCα), and glyceraldehyde‐3‐phosphate dehydrogenase (IgG_1_ κ GAPDH) were purchased from Santa Cruz Biotechnology (Dallas, TX, USA). Primary antibody for human glucose transporter 2 (IgG Glut 2) was obtained from Sigma (St. Louis, MO, USA). Secondary antibodies were from Cytiva (Marlborough, MA, USA). Western blot materials were from Bio‐Rad (Hercules, CA, USA). Linagliptin (highly potent and selective competitive inhibitor of DPP IV, >98% pure) from AdipoGen (San Diego, CA, USA). Other materials were from Sigma (St. Louis, MO, USA), unless noted otherwise.

### 2.2 Selection of Peptides From Digested AB7S and S7S Protein and Prediction of Peptide Pharmacokinetic Behavior by In Silico pkCSM Analysis

Peptides from digested AB7S and S7S proteins were sequenced and evaluated [20]. Peptides were selected based on the following criteria: (1) <13 amino acids in length, (2) molecular mass <3 kDa, (3) hydrophobicity (∼7–8 kcal/mol), (4) in silico bioactivity and the biochemical DPP VI inhibitory potential occurrence frequency in the peptide, and (5) the binding energy from in silico molecular docking. The pure peptides were purchased and tested in healthy and insulin‐resistant HepG2 cells.

Pharmacokinetic and toxicity properties of the selected peptides were predicted using the pkCSM tool [39].

### 2.3 Luminescence‐Based DPP IV Inhibition Biochemical Assay, and Peptide Interaction Assessment in the Cell‐Free System

The luminescence DPPIV‐GLO Protease assay (G8351, Promega, Madison, WI) was performed to measure the DPP IV inhibitory capacity of selected AB7S peptides and S7S peptides [20]. Percent inhibition was calculated for each sample. Results were presented as percent inhibition per log (µg peptide/mL). The inhibitory concentration (IC) values of the peptides were calculated using Origin Pro software (Version 2021, OriginLab Corporation, Northampton, MA, USA).

A peptide interaction assessment for VP and IPA was conducted using the DPP IV inhibitory assay. Two concentrations (IC_25_ value and IC_50_ value obtained from the DPP IV inhibition assay) were used for each peptide. Peptides were combined in a 1:1 ratio. The assessment was performed with at least three replicates per treatment and concentration. Synergy calculations were performed using SynergyFinderPlus [40]. Docking of VP and IPA with the DPP IV enzyme was performed as indicated before, and more specific details could be found in the previous publication from our group [20]. Briefly, one hundred runs were performed by AutoDock Tools (Scripps Research, San Diego, CA) to identify the best binding conformation based on the lowest binding energy. The DPP IV protein structure was modified by removing water molecules, and adding polar hydrogens and Kollman charges [20].

### 2.4 Cell Culture, Treatment Conditions, Cell Viability, and DPP IV Enzyme Activity

Human liver HepG2 cells (HB‐8065) were obtained from the American Type Culture Collection (ATCC, Manassas, VA, USA) and cultured in Eagle's Minimum Essential Medium (EMEM) supplemented with 10% fetal bovine serum (FBS) and 1% penicillin‐streptomycin. Cells were maintained at 37°C in 5% CO_2_/95% air using a CO_2_ Jacketed Incubator (NuAIRE DH Autoflow, Plymouth, MN).

HepG2 cells were seeded in either 96‐well plates or 6‐well plates for 24 h. For the healthy cell model (NG), cells were incubated in a medium with a healthy/normal glucose level (5.5 mM glucose), and no stimulation was performed. For the insulin‐resistant cell model (HG), cells were stimulated according to the methods used in a previous publication [20]. In brief, cells were treated for 24 h using 25 mM glucose with 100 nM insulin to stimulate insulin resistance in the HepG2 cells. After seeding or insulin‐resistant stimulation, the cells were treated with selected pure AB7S peptides, selected pure S7S peptides, peptide Asp‐Leu‐Asp‐Val (DLDV; peptide with no DPP IV inhibitory capacity), or 1 nM linagliptin (DPP IV inhibitor) for 24 h. As this study focused on the DPP IV inhibition capacity of the peptides and how such inhibition affects the insulin signaling in the cells, we used linagliptin (the most potent DPP IV inhibitor) as the positive control. Treated cells were used to either perform cell viability assays or were collected, centrifuged, and stored at −80°C until being used.

### 2.5 Cell Viability Assay

Three concentrations (17.9–1790 µM) of selected pure AB7S peptides and selected pure S7S peptides were tested. Linagliptin (1 nM) was used as a positive control. Treated cells were tested for viability by the MTS‐cell viability assay using the CellTiter 96 Aqueous One Solution Proliferation assay according to the manufacturer's instructions (Promega Corporation, Madison, WI, USA).

### 2.6 Luminescence‐Based DPP IV Assay in the Cell‐Based System

DPP IV inhibition was measured using the luminescence DPPIV‐GLO Protease assay (G8351, Promega, Madison, WI) [20]. In brief, treated cells were incubated with three concentrations of selected pure AB7S peptides or selected pure S7S peptides for 24 h, and linagliptin (1 nM) was used as a positive control. Treated cells were collected and stored at −80°C for no more than one week until being used for the DPPIV‐GLO protease assay. Percent inhibition was calculated, and results were presented as percent inhibition per log (µg peptide/mL). The IC values of the peptides were calculated using Origin Pro software (Version 2021, OriginLab Corporation, Northampton, MA, USA). The IC_50_ values of the most potent AB7S and S7S peptides were used as the treatment concentration in the in vitro experiments.

### 2.7 Immunoassay Proteomics

Cells treated with VP (9.58 µM) and the untreated cells were tested using immunoassay proteomics. The IC_50_ value of VP obtained from the luminescence‐based DPP IV inhibition biochemical assay was used as the treatment concentration. Cells were treated as described in Section 2.4. An insulin receptor microarray was performed according to the manufacturer's instructions to test the protein expression of markers in the insulin signaling pathway (Insulin Receptor Phospho Antibody Array, PIG219, Full Moon BioSystem, Sunnyvale, CA, USA). Array signals were visualized with a 20× objective using a Widefield Fluorescence Microscope (Carl Zeiss AG, Germany) and were analyzed as previously described [41]. Student's *t*‐test was used to assess the differences between the VP treatment and the negative control.

### 2.8 Hepatic Protein Expression of Related Markers by Western Blot

Cells treated with VP (9.58 µM), IPA (7.86 µM), DLDV (1790 µM), or linagliptin (1 nM) and untreated cells were used for western blot [20]. In brief, membranes were incubated with conjugated antibodies for human IRS‐1 (sc‐8038 HRP, 1:100) and Akt‐1 (sc‐5298 HRP, 1:100), or ACCα (sc‐137104 HRP, 1:100) overnight at 4°C and washed. Membranes were also incubated with unconjugated primary antibodies for human Glut 2 (07‐1402, 1:1000), then washed and probed with secondary sheep anti‐mouse and donkey anti‐rabbit antibodies (NA931V and NA934V, 1:2500, 2 h, room temperature; Cytiva, Marlborough, MA, USA). GAPDH (sc‐25778 HRP or sc‐47724, 1:4000) was used as the protein loading control and was used to evaluate the relative expression of each protein. Clarity Western ECL Substrate (Bio‐Rad, Hercules, CA, USA) and ImageQuant 800 System (GE Healthcare, Buckinghamshire, UK) were used for imaging.

### 2.9 Glut 2 Expression by Immunocytochemical Fluorescence Confocal Microscopy

The expression of Glut 2 due to the peptide treatment (VP, 9.58 µM) was investigated in HepG2 cells seeded in Ibidi imaging dish (Ibidi, Fitchburg, WI, USA). Cells were treated as described in Section 2.4. The positive control (1 nM linagliptin) was also tested. The immunocytochemical fluorescence confocal microscopy was performed according to a previous procedure [42]. In brief, cells were fixed, permeabilized, blocked, and incubated with Glut 2 antibodies (07‐1402, 1:1000) overnight at 4°C. Cells were then washed twice, incubated with anti‐rabbit IgG secondary antibody (A‐11012; 1:200) for 2 h in the dark, cured with ProLong Gold Antifade Reagent with DAPI (4′,6‐diamidino‐2‐phenylindole), and stored at 4°C until imaging. Cell images were obtained with 63× oil immersion objective (Glut 2: excitation wavelength: 557 nm, emission wavelength: 572 nm; DAPI: excitation wavelength: 353 nm; emission wavelength: 465 nm) using a Carl Zeiss LSM 900 Laser Scanning Microscope (Carl Zeiss AG, Germany).

### 2.10 Statistical Analysis

Data was analyzed using Origin (Pro) 2021 software (OriginLab Corporation, Northampton, MA, USA). One‐way analysis of variance (ANOVA) and a post hoc Tukey's test were used to determine the significant differences among the samples. A *p* value < 0.05 was considered statistically significant. All analyses were tested and validated for accuracy and precision before running the tested samples.

## 3 Results

### 3.1 Selected Peptides From Digested AB7S and S7S Protein and Their In Silico Pharmacokinetic Properties

Peptides VP, PM, and FNE (Figure S1) sequenced from digested AB7S protein and peptides LLS, IPA, and VP from digested S7S protein were selected by their high relative abundance on the chromatogram (PM: 0%–30%, FNE: 0%–30%, LLS: >50%, IPA: >50%, VP: >50%, respectively) and by their absorption, distribution, metabolism, excretion, and toxicity properties (Table 1). In brief, the intestinal absorption of the peptides PM, VP, IPA, LLS, FNE, and DLDV was 84.8%, 68.5%, 34.3%, 15.8%, 0%, and 0%, respectively. The blood‐brain barrier permeability of the peptides ranged from –1.2 to –0.5, while the central nervous system permeability of the peptides ranged from –4.1 to –3.1. No AMES toxicity and skin sensitization were found for all the peptides. For the hepatotoxicity test, peptides PM, VP, and IPA were not toxic to the liver, while peptides FNE, LLS, and DLDV were found to be toxic. None of the peptides were human ether‐a‐go‐go gene (hERG) I and hERG II inhibitors. In silico pharmacokinetic prediction suggested peptides VP, and IPA have good absorptivity and were safe to consumers.

**TABLE 1:** Pharmacokinetic and toxicity properties of adzuki bean and soybean peptides using the pKCSM tool.

	FNE	PM	VP	LLS	IPA	DLDV
Absorption						
Water solubility (log mol/L)	−2.5	−0.8	−1.2	−2.1	−2.0	−2.7
Caco2 permeability (log Papp in 10^−6^ cm/s)	−0.3	0.5	0.7	−0.4	−0.3	−1.4
Intestinal absorption (Human) (% Absorbed)	0	84.8	68.5	15.8	34.3	0
Skin Permeability (log Kp)	−2.7	−3.0	−2.7	−2.7	−2.7	−2.7
P‐glycoprotein substrate	Yes	Yes	No	Yes	No	No
P‐glycoprotein I inhibitor	No	No	No	No	No	No
P‐glycoprotein II inhibitor	No	No	No	No	No	No
Distribution						
Volume of distribution (human) (log L/kg)	−1.1	0.1	−0.5	−1.0	−0.7	−1.2
Fraction unbound (human) (Fu)	0.6	0.7	0.8	0.7	0.8	0.6
Blood‐brain barrier permeability (log BB)	−0.9	−0.8	−0.5	−0.8	−0.8	−1.2
Central nervous system permeability (log PS)	−4.1	−3.4	−3.1	−3.9	−3.6	−3.5
Excretion						
Total clearance	0.7	0.8	0.4	0.2	0.5	−0.6
Renal organic cation transporter 2 substrate	No	No	No	No	No	No
Toxicity						
AMES toxicity	No	No	No	No	No	No
Max. tolerated dose (human) (log mg/kg/day)	0.8	0.5	1.1	1.2	1.1	0.5
Human ether‐a‐go‐go gene I inhibitor	No	No	No	No	No	No
Human ether‐a‐go‐go gene II inhibitor	No	No	No	No	No	No
Oral Rat Acute Toxicity (LD50) (mol/kg)	2.1	2.3	1.7	1.4	1.8	2.4
Oral Rat Chronic Toxicity (LOAEL) (log mg/kg body weight/day)	3.3	1.1	1.5	2.8	2.5	3.4
Hepatotoxicity	Yes	No	No	Yes	No	Yes
Skin Sensitization	No	No	No	No	No	No
*T. pyriformis* toxicity (log ug/L)	0.3	0.1	0.3	0.3	0.3	0.3
Minnow toxicity (log mM)	4.4	2.5	2.9	2.9	3.2	6.5

*Note*: Pharmacokinetic and toxicity properties of adzuki bean and soybean peptides using the pKCSM tool [Accessed September 23, 2024].

**Metabolism**: Peptides were not Cytochrome P450 (CYP) 2D6 substrates, CYP3A4 substrates, CYP1A2 inhibitors, CYP2C19 inhibitors, CYP2C9 inhibitors, CYP2D6 inhibitors, or CYP3A4 inhibitors.

### 3.2 AB7S and S7S Individual Peptides Exhibited DPP IV Enzyme Inhibitory Potential but Had an Antagonistic Interaction in the Cell‐Free System

**FIGURE 1:**
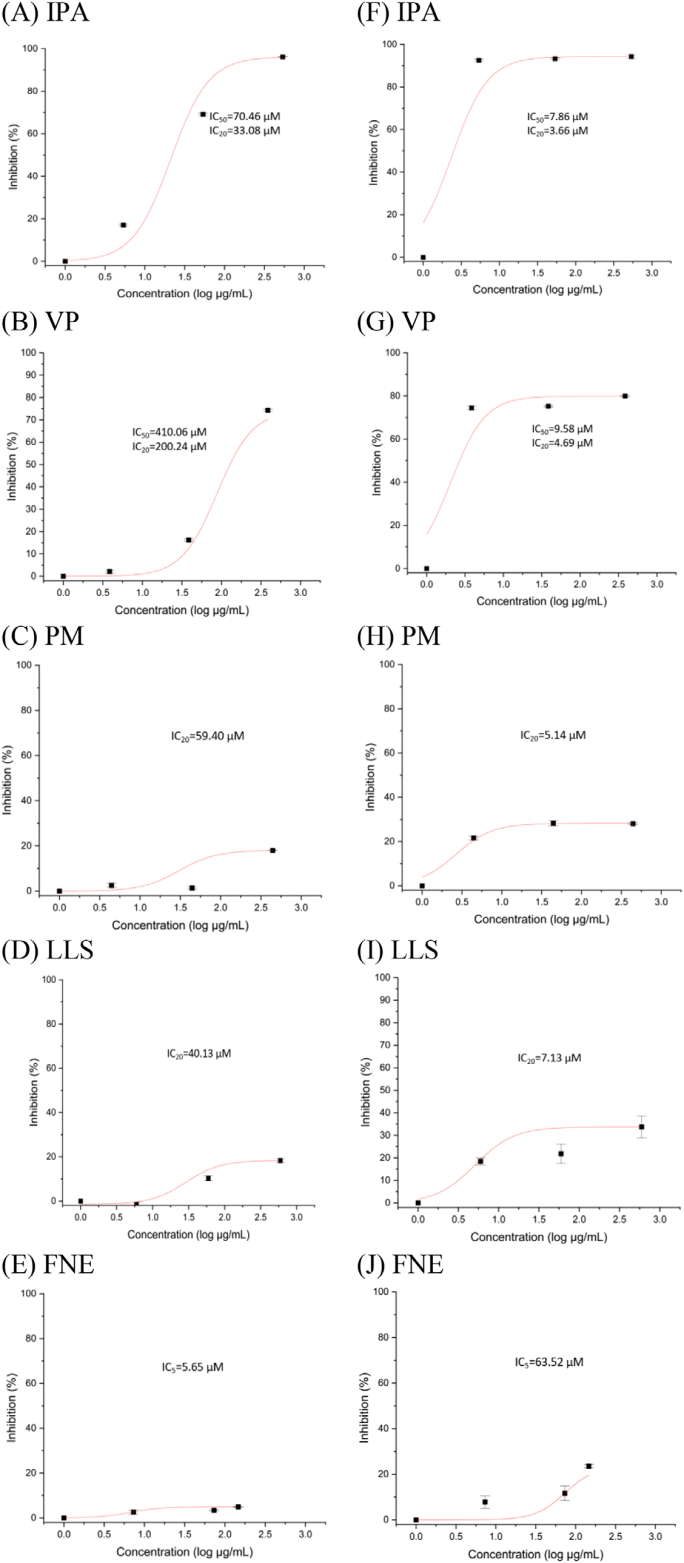
Dose‐response curves of adzuki bean β‐vignin peptides (PM, FNE, VP) and soybean β‐conglycinin peptides (IPA, LLS, VP) on the inhibition of human DPP IV activity. (A–E) Dose‐response curves of the peptides using purified human DPP IV enzyme, and (F–J) dose‐response curves of the peptides using HepG2 cells. Inhibitory concentration (IC) values were calculated from the antilog of the x‐axis value at the inflection point of each sigmoid‐curve fit. Results are presented as mean ± standard error of ≥3 independent experiments. IPA, peptide Ile‐Pro‐Ala; VP, peptide Val‐Pro; PM, peptide Pro‐Met; LLS, peptide Leu‐Leu‐Ser; FNE, peptide Phe‐Asn‐Glu.

**FIGURE 2:**
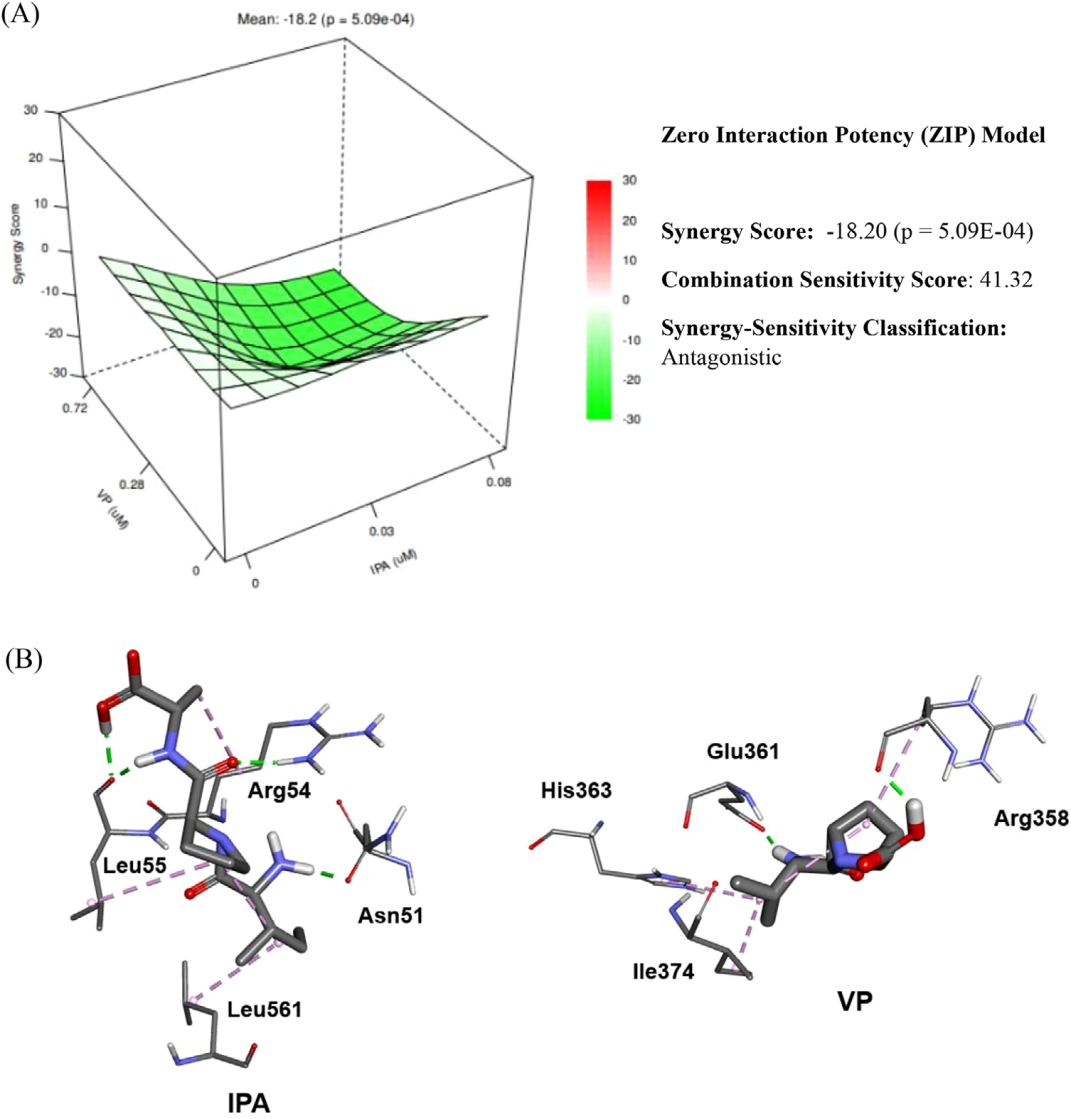
Effect of peptide‐peptide interaction of VP and IPA on DPP IV inhibition. (A) Synergy score, combination sensitivity score, and synergy‐sensitivity classification of VP and IPA combinations based on the Zero Interaction Potency (ZIP) model on DPP IV inhibition in the cell‐free system. Results are presented as mean ± standard error of ≥3 independent experiments. (B) Structures of VP and IPA and their binding sites on dipeptidyl peptidase IV (PDB ID: 1RWQ).

In the dose‐response experiments conducted using the purified human DPP IV enzyme (cell‐free system), IPA (IC_50_, 70.46 µM; IC_20_, 33.08 µM) was the most potent peptide in inhibiting DPP IV activity, followed by VP (IC_50_, 410.06 µM; IC_20_, 200.24 µM), LLS (IC_20_, 40.13 µM), PM (IC_20_, 59.40 µM), and FNE (IC_5_, 5.65 µM) (Figure 1A–E). In the interaction study, combining IPA and VP at a 1:1 ratio had an antagonistic effect on DPP IV inhibition and a combination sensitivity score of 41.32 (Figure 2A). Docking results indicated that VP and IPA are bound to the same DPP IV binding site (Figure 2B). Peptides VP and IPA exhibited the greatest DPP IV enzyme inhibitory potential.

### 3.3 AB7S and S7S Peptides Were Not Cytotoxic and Exhibited DPP IV Enzyme Inhibitory Potential

The cell viability in the healthy state (5.5 mM glucose, NG) was >80% in the HepG2 cells in response to the peptide treatments when compared to the untreated cells (Figure S2A–E). In the dose‐response in vitro experiments conducted using the HepG2 cell culture, IPA (IC_50_, 7.86 µM; IC_20_, 3.66 µM) was the most potent peptide in inhibiting DPP IV activity, followed by VP (IC_50_, 9.58 µM; IC_20_, 4.69 µM), PM (IC_20_, 5.14 µM), LLS (IC_20_, 7.13 µM), and FNE (IC_5_, 63.52 µM) (Figure 1F–J). Peptides VP and IPA were not cytotoxic and exhibited the greatest DPP IV enzyme inhibitory potential.

### 3.4 Protein Microarray Showed That VP Enhanced the Insulin Signaling Pathway in HepG2 Cells in the Healthy State

The VP (9.58 µM) treatment significantly changed (*p* < 0.05) the phosphorylation pattern of eight markers related to the insulin signaling pathway; Table 2 presents the phosphorylated/non‐phosphorylated ratio (fold change related to the untreated control). The phosphorylation ratios of Akt, AKT1 substrate 1 (Akt1S1), AMP‐activated protein kinase catalytic subunit alpha‐1/2 (AMPK1/2), SHC‐transforming protein 1 (Shc), mechanistic target of rapamycin (mTOR), protein phosphatase 2 alpha (PP2A‐α), and insulin receptor (IR) were significantly increased (*p* < 0.05) after the VP treatment. In contrast, the phosphorylation ratio of the IRS‐1 was significantly decreased (*p* < 0.05) by the treatment. These changes suggested that the insulin signaling pathway was enhanced.

**TABLE 2:** Protein microarray results showed the fold changes of insulin pathway‐related markers in HepG2 cells treated with the peptide VP compared to untreated controls in the healthy state.

Marker	Indication of the changes in phosphorylation ratio	Phosphorylated/Non‐phosphorylated ratio (Fold Change related to the untreated control, *p* < 0.05)
Akt (AKT serine/threonine kinase)	Increased phosphorylation ratio of the protein stimulates the insulin signaling pathway	3.9^*^
mTOR (Mechanistic target of rapamycin)	Increased phosphorylation ratio indicates activation of the insulin signaling pathway	3.6^*^
AMPK1/AMPK2 (AMP‐activated protein kinase catalytic subunit alpha‐1/2)	Increased phosphorylation ratio inhibits fatty acid synthesis	3.5^*^
Shc (SHC‐transforming protein 1)	Increased phosphorylation ratio indicates activation of the insulin signaling pathway	3.2^*^
PP2A‐α (Protein phosphatase 2 alpha)	Increased phosphorylation ratio indicates activation of the insulin signaling pathway	2.6^*^
IR (Insulin receptor)	Increased phosphorylation ratio stimulates the insulin signaling pathway	1.7^*^
Akt1S1 (AKT1 substrate 1)	Increased phosphorylation ratio of this substrate stimulates the insulin signaling pathway	1.3^*^
IRS‐1 (Insulin receptor substrate 1)	Increased phosphorylation ratio inhibits the insulin signaling pathway	0.2^*^

*Note*: Protein microarray results showed the fold changes of insulin pathway‐related markers in HepG2 cells treated with the peptide VP compared to untreated controls in the healthy state. Fold change was calculated relative to the untreated control. Fold change lower than 1 indicated a decrease in expression, and fold change higher than 1 indicated an increase in expression. Asterisks * represent statistically significant differences at *p* < 0.05. Statistically significant fold changes were presented.

### 3.5 Western Blot Results Showed That Peptides From Digested AB7S and S7S Protein Modulated Insulin Signaling‐ and Glucose Uptake‐Related Markers but Not Lipogenic‐Related Markers in the Healthy State and the Insulin‐Resistant State

**FIGURE 3:**
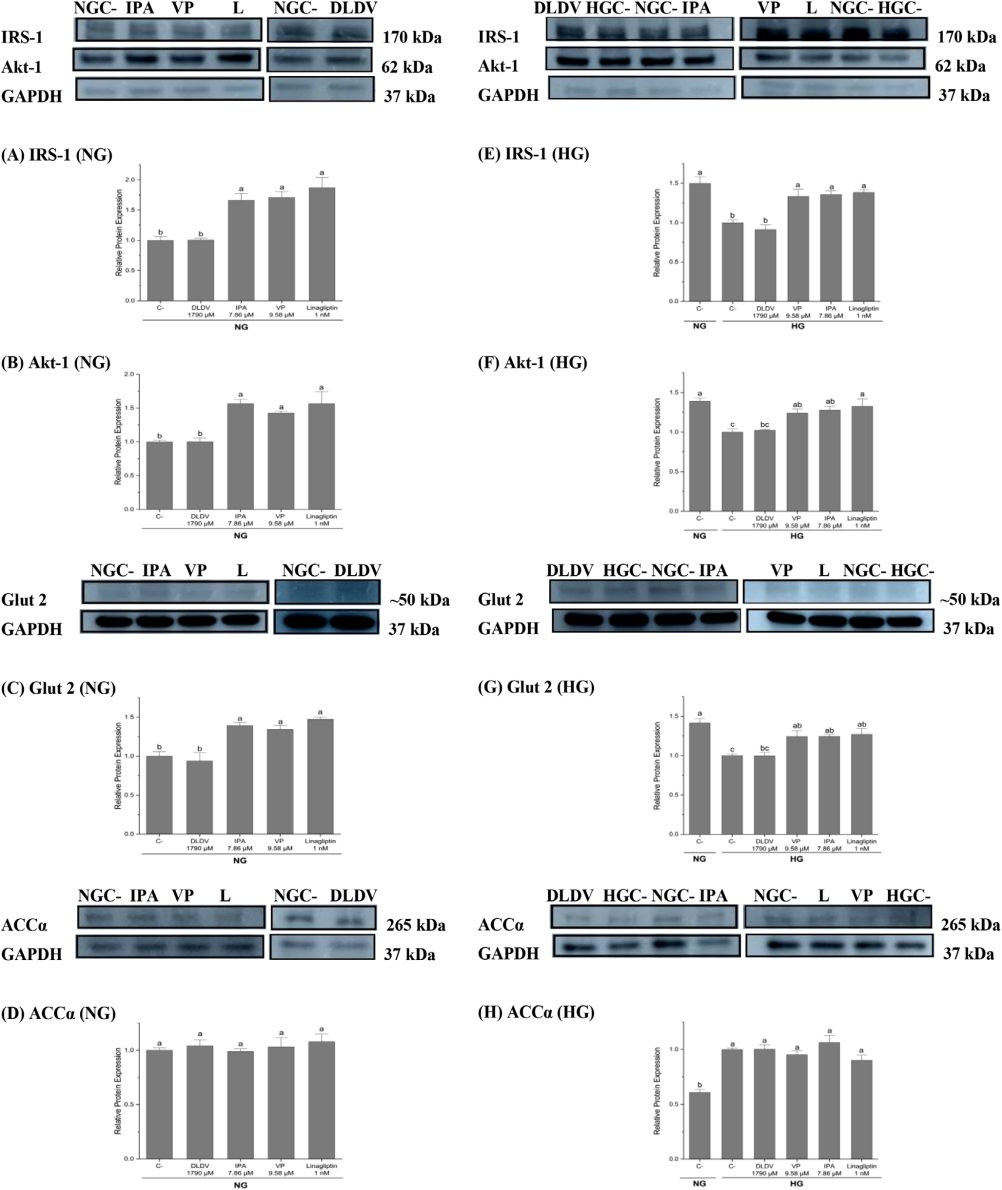
Protein expression of insulin signaling‐, glucose uptake‐, and lipogenic‐related markers in HepG2 cells in response to adzuki bean and soybean peptides and linagliptin (L) in the healthy state (NG, 5.5 mM glucose) and insulin‐resistant state (HG, 25 and 100 nM insulin). (A) IRS‐1 expression, (B) Akt‐1 expression, (C) Glut 2 expression, and (D) ACCα expression in treated cells treated in NG. (E) IRS‐1 expression, (F) Akt‐1 expression, (G) Glut 2 expression, and (H) ACCα expression in treated cells in HG. Relative protein expression was normalized to non‐treated cells in the respective NG or HG states. A representative image of the Western blot is shown on top of the respective graph. ANOVA with Tukey's post hoc test were performed. Letters indicate a significant difference at *p* < 0.05. Results are presented as mean ± standard error of ≥3 independent experiments.

Peptides from digested AB7S and S7S changed the markers related to insulin signaling and hepatic glucose uptake during the healthy and insulin‐resistant states. In the healthy state, the protein expressions of IRS‐1, Akt‐1, and Glut 2 in HepG2 cells treated with VP (9.58 µM), IPA (7.86 µM), or linagliptin (1 nM) were significantly higher (IRS‐1: >50% for all treatments; Akt‐1: >25% for all treatments; Glut 2: >25% for all treatments) than that in the untreated controls (*p* < 0.05) (Figure 3A–C). However, no changes were found in the expression of the lipogenic marker ACCα (*p* > 0.05) (Figure 3D).

In the insulin‐resistant state, the untreated cells significantly reduced the protein expression of IRS‐1 (33%), Akt‐1 (28%), and Glut 2 (29%) in comparison to the healthy state, while increasing the protein expression of ACCα (64%). Yet, treating the cells, in the insulin‐resistant state, with VP and IPA significantly increased the protein expression of IRS‐1, Akt‐1, and Glut 2 (IRS‐1: >25% for both treatments; Akt‐1: >25% for both treatments; Glut 2: >25% for both treatments, *p* < 0.05) (Figure 3E–G). Like the findings in the healthy state, the ACCα protein expression in both the treated and untreated cells was not different (*p* > 0.05) in the insulin‐resistant state (Figure 3H). Overall, no differences were observed in the protein expression of any of the markers between cells treated with DLDV (1790 µM) and the untreated control (*p* > 0.05) in both the healthy and insulin‐resistant states. These results suggested that the peptides selected, except for DLDV, played similar roles in modulating the markers in both healthy and insulin‐resistant states.

### 3.6 Confocal Microscopy Results Showed That VP Stimulated the Expression of Glut 2 in HepG2 Cells in Healthy and Insulin‐Resistant States

**FIGURE 4:**
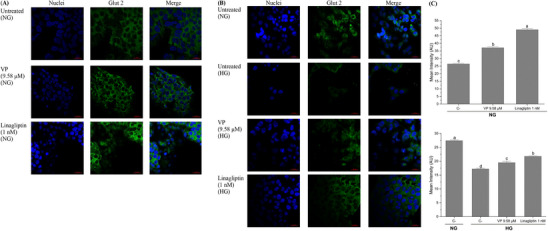
Immunocytochemical fluorescence confocal microscopy on Glut 2 protein expression in HepG2 cells treated with VP (9.58 µM) or linagliptin (1 nM) (A) the healthy state (NG, 5.5 mM glucose) and (B) in the insulin‐resistant state (HG, 25 and 100 nM insulin). Glut 2 expression in cells was imaged using an objective 63× oil immersion (20 µm scale). (C) Quantification of Glut 2 expressed as average intensity over their respective area (AU) per cell. ANOVA with Tukey's post hoc test were performed. Letters indicate a significant difference at *p* < 0.05. Results are presented as mean ± standard error of two independent fields of view from two independent cellular treatment replicates. The panel to the left represents DAPI‐stained nuclei (blue), the panel in the middle shows the response to the Glut 2 expression (green), and the third panel represents the merger of both.

**FIGURE 5:**
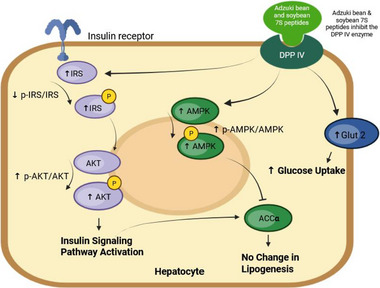
Proposed mechanism of peptides identified in digested adzuki bean β‐vignin (adzuki bean 7S) and soybean β‐conglycinin (soybean 7S) on glucose uptake, insulin signaling, and lipogenic pathways. DPP IV, dipeptidyl peptidase IV; IRS, insulin receptor substrate; p‐IRS, phosphorylated IRS; AKT, protein kinase B; p‐AKT, phosphorylated AKT; Glut 2, glucose transporter type 2; AMPK, AMP‐activated protein kinase; p‐AMPK, phosphorylated AMPK; ACCα, acetyl‐CoA carboxylase α.

Immunocytochemical fluorescence confocal microscopy confirmed that cells treated with VP (9.58 µM) or linagliptin (1 nM) had a significantly higher (36% for both treatments, *p* < 0.05) Glut 2 fluorescence emission than the untreated control in the healthy state (Figure 4). The insulin‐resistant HepG2 cells had a significantly lower (*p* < 0.05) Glut 2 fluorescence emission in comparison to the healthy HepG2 cells; however, insulin‐resistant cells treated with VP or linagliptin had a significant increase (>14% for both treatments) in Glut 2 fluorescence emission compared to the untreated insulin‐resistant cells. These results confirmed VP's ability to modulate the expression of Glut 2. Based on the results obtained, Figure 5 presents the potential mechanism of AB7S and S7S peptides in modulating the insulin signaling and glucose uptake in the liver.

## 4 Discussion

To date, no other studies have reported the pharmacokinetic properties of the peptides sequenced and characterized from AB7S and S7S. Based on the in silico pkCSM results, PM from AB7S, IPA from S7S, and VP from both proteins have the potential to be absorbed via the intestine, which is the main absorption site for orally administered medications [43]. Their intestinal absorption was >30%, suggesting the peptides could be absorbed via the human intestine [44]. PM seemed to be the easiest to be absorbed, followed by VP and IPA (84.8%, 68.5%, and 34.3%, respectively). All of the peptides were poorly distributed to the brain as they all had a low permeability for the blood‐brain barrier, which protects the brain against harmful substances [45], and they were not able to penetrate the central nervous system. This minimizes the unintended neurological effects of the peptides [46, 47]. PM, VP, and IPA were not predicted to be related to hepatotoxicity, skin sensitization, and fatal ventricular arrhythmia. Negative test results from the Ames toxicity test were found, suggesting they did not have mutagenic and carcinogenic potential [48]. Furthermore, the three peptides had a high maximum tolerated dose, suggesting that consuming these peptides at higher doses was less likely to cause adverse effects that could be hazardous to humans, which was confirmed by ToxinPred [49, 50]. Since both IPA and VP inhibit the DPP IV at the same binding site, the two peptides might compete with each other, which contributes to their antagonistic relationship [20].

There are limited studies investigating the efficacy of AB7S peptides and S7S peptides in DPP IV inhibition. Therefore, we conducted dose‐response experiments using both the HepG2 cells (cell‐based system) and the purified human DPP IV enzyme (cell‐free system). The direct use of human purified DPP IV enzyme in determining the inhibitory efficacy of the peptides provided insight into how the peptides affected the activity of the enzyme; however, this system lacked the complexity and physiological relevance of the HepG2‐based system [51]. Therefore, we used the IC_50_ values of the most potent AB7S and S7S peptides obtained in the HepG2 system as the treatment concentration in the in vitro experiments.

AB and soybean peptides might play a role in the regulation of the upstream regulators of the DPP IV enzyme. Cytokines (such as IL‐12 and TNF‐α) and transcription factor (NFκB) modulated DPP IV activity [52, 53]. Shi et al. [54] found that AB peptide Lys‐Gln‐Ser‐1 inhibited NF‐κB. Likewise, Kim et al. [55] reported that soybean peptides downregulated the levels of NFκB. Also, soybean oligopeptides inhibited cytokine induction [56]. Thus, it is possible that the selected AB and soybean peptides could also inhibit the DPP IV activity via modulating the DPP IV upstream regulators, in addition to directly inhibiting the DPP IV enzyme. Additional studies are needed to investigate this relationship.

Previous studies reported that linagliptin regulates the blood glucose level via inhibiting the DPP IV activity and modulating the insulin signaling pathway [57, 58]. Therefore, linagliptin was used as the positive control in our study. The insulin signaling pathway is crucial for maintaining glucose homeostasis [59, 60, 61]. It also plays an important role in lipid metabolism [59]. Results showed that VP improved T2D‐related markers. IRS‐1 and Akt‐1 are the main markers of the insulin signaling pathway, and their phosphorylation ratio can indicate the activation or inhibition of the insulin signaling pathway [62, 63, 64]. In the healthy state, treating HepG2 cells with VP significantly increased the phosphorylated/non‐phosphorylated ratio of IR, Akt, Akt1S1, and mTOR while significantly reducing the phosphorylated/non‐phosphorylated ratio of IRS‐1. This confirmed that VP played a role in activating the insulin signaling pathway.

The activated insulin signaling pathway also plays a role in fatty acid synthesis via upregulating the expression of key lipogenic enzymes, such as fatty acid synthase and SREBP1 [65, 66]. The activated insulin signal pathway and resulting activation of mTOR upregulate ACCα, the rate‐limiting enzyme for lipogenesis, and lipogenesis [67, 68, 69, 70]. On the other hand, in vitro results also showed an increase in AMPK phosphorylation ratio, suggesting a downregulation of lipogenesis [71, 72, 73]. Previous studies reported that the insulin signaling pathway counteracts the AMPK signaling pathway [74]. The insulin signaling pathway favors anabolic processes in the cells, while the AMPK pathway favors catabolic processes in the cells [75, 76]. The finding that treating HepG2 cells with VP or IPA did not affect the expression of ACCα suggested that the upregulation of lipogenesis from the activated IRS‐1/Akt‐1/mTOR pathway counteracted the downregulation effect from the activated AMPK pathway. Therefore, more research studies examining the interaction between the insulin‐AKT signaling and the AMPK pathways are needed.

Glut 2 is one of the most important proteins in the liver that contributes to hepatic glucose uptake. More than 97% of the glucose transporters in the liver are Glut 2 proteins, and reduction of Glut 2 activity inhibits glucose uptake [77, 78]. Increased HepG2 Glut 2 protein expression by IPA and VP suggested the potential of these peptides in upregulating hepatic glucose uptake. VP was >100 times more powerful in increasing the protein expression of Glut 2 than the digested adzuki bean β‐vignin protein based on previous data [20].

Insulin‐resistant cells were used to determine the effect of these peptides on T2D‐related markers. Insulin resistance is associated with decreased IRS‐1 and Glut 2 protein expression in the liver [79, 80, 81]. The peptides tested significantly increased the protein expression of IRS‐1, Akt‐1, and Glut2, in comparison with the control, suggesting that peptides present in digested AB7S and S7S may improve glucose‐related outcomes during insulin resistance states, like the effect observed in the AB7S digest [20]. Similar to the findings in the healthy state, no changes were observed in the insulin‐resistant state regarding the protein expression of ACCα, suggesting that similar underlying mechanisms may contribute to lipogenesis regulation. Future studies will investigate markers from other glucose metabolism‐related pathways to better understand how VP regulates hepatic glucose metabolism and helps manage T2D‐related outcomes.

This study had some limitations. The intestinal absorption of the peptides was based on the computational pkCSM prediction, and experimental pharmacokinetic data were needed to verify the actual absorption potential of these peptides. Moreover, this study used a monoculture system, which could not replicate the in vivo hormonal and metabolic interactions and had a limited representation of human liver insulin signaling and generalizability. Therefore, animal studies (and studies using hepatic cells isolated from the animals) are needed to explore the anti‐diabetic potential of AB7S peptides in a greater physiological relevance. In addition, peptides might degrade when applied to the HepG2 cells [82, 83]; therefore, peptide stability assessments are needed. Although the IC_50_ values for the peptides in inhibiting the DPP IV enzyme were tested in this study, adjustments to the IC_50_ values are needed to obtain the maximum plasma concentration of the peptides [84, 85]. Therefore, more studies are needed to translate the in vitro findings to the effective concentrations in vivo.

In conclusion, this study identified the functional peptides contributing to the antidiabetic potential of AB7S and examined the underlying mechanisms in greater depth. Of the five pure peptides tested, IPA and VP were the two most potent peptides in inhibiting DPP IV. These peptides were not cytotoxic to the cells at almost 2 mM and effectively enhanced the insulin signaling pathway and the hepatic glucose uptake. In vitro cell culture experiments confirmed the DPP IV inhibitory and antidiabetic potential of these peptides. Both peptides were effective in modulating markers involved in the insulin signaling pathway and hepatic glucose uptake in both healthy and insulin‐resistant states. Therefore, peptides from digested AB7S and S7S have health‐benefiting properties via their potential in inhibiting the DPP IV enzyme and enhancing insulin signaling and hepatic glucose uptake. This study provided the scientific bases for further in vivo long‐term glucose control studies and helped increase the formulation potential of healthier AB‐plant‐based functional food ingredients to support T2D management.

## Conflicts of Interest

The authors declare no conflict of interest.

## Supporting information




**Supporting File 1**: mnfr70285‐sup‐0001‐SuppMat.docx

## Data Availability

The data that support the findings of this study are available from the corresponding author upon reasonable request.
