# Supplementary material for: Peptides From Adzuki Bean and Soybean Improved Insulin‐AKT Signaling‐Related Pathways in Healthy and Insulin‐Resistant States in Human Liver Cells
Source: Mol Nutr Food Res. 2025 Oct 9;69(24):e70285. doi: 10.1002/mnfr.70285 (PMC12700049; doi:10.1002/mnfr.70285)
Supplement: Supplementary file 1 — Supporting File 1: mnfr70285‐sup‐0001‐SuppMat.docx [file MNFR-69-e70285-s001.docx]

| (A) IPA  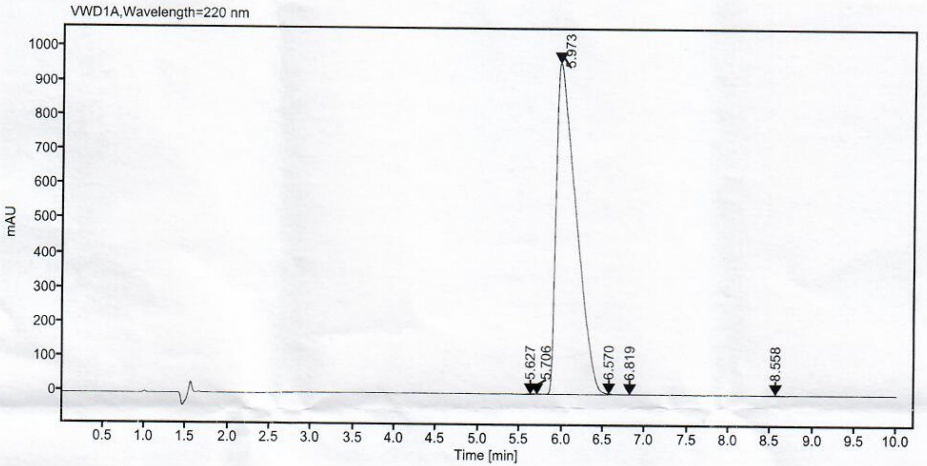 | (B) VP  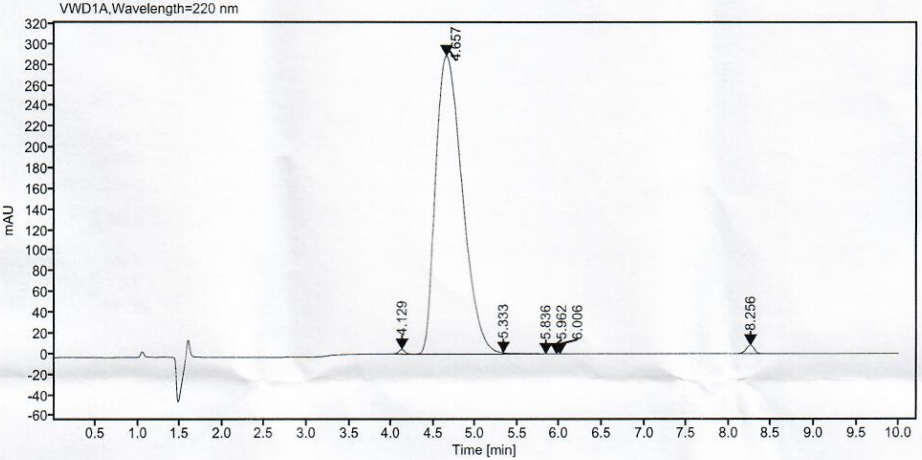 |
| --- | --- |
| (C) PM  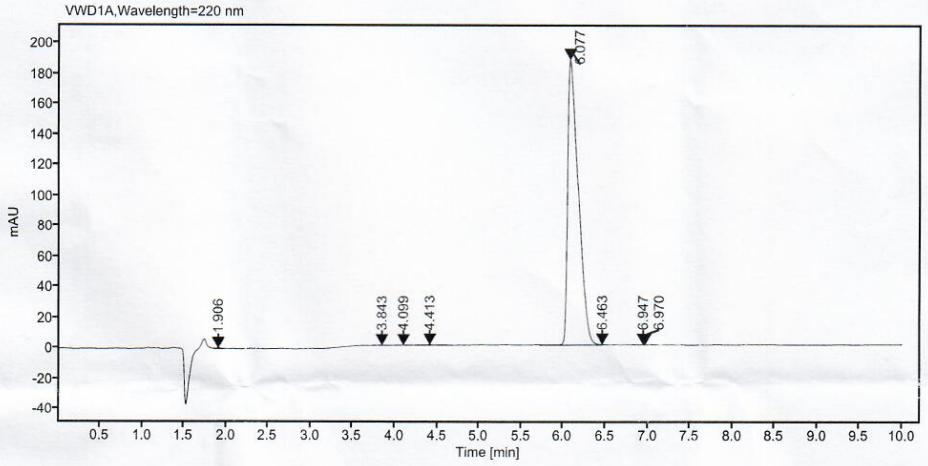 | (D) LLS  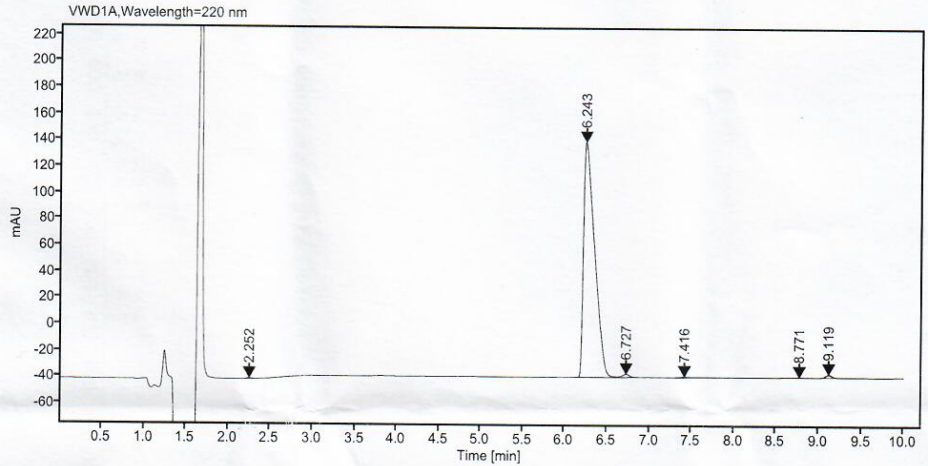 |
| (E) FNE  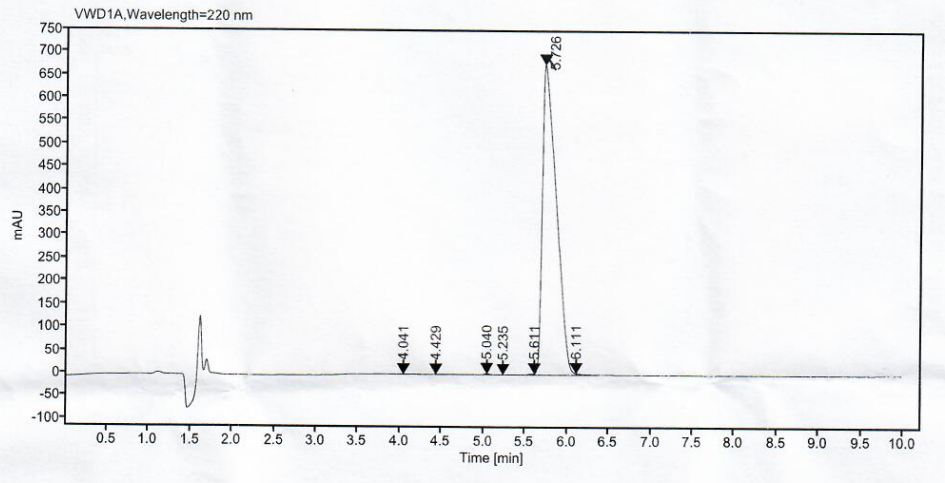 | (F) DLDV  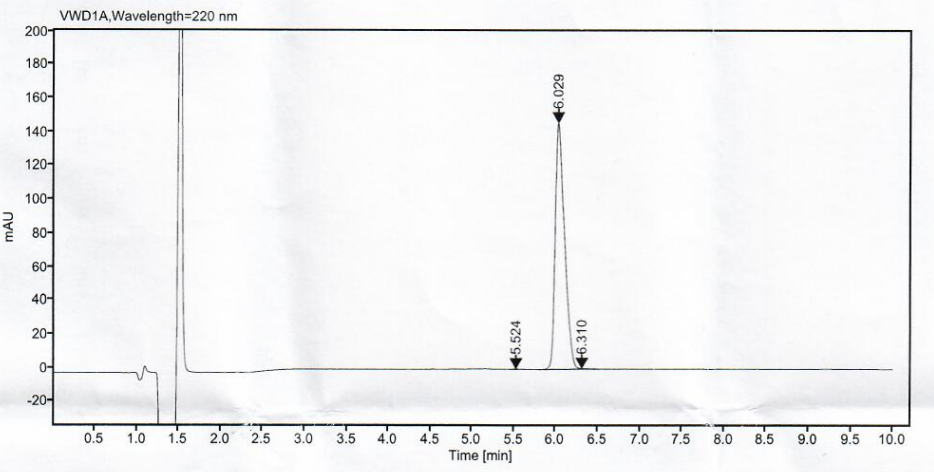 |

**Supplementary Figure 1.** RP-HPLC of the synthesized peptides. IPA, peptide Ile-Pro-Ala; VP, peptide Val-Pro; PM, peptide Pro-Met; LLS, peptide Leu-Leu-Ser; FNE, peptide Phe-Asn-Glu; DLDV, peptide Asp-Leu-Asp-Val.

| (A) IPA  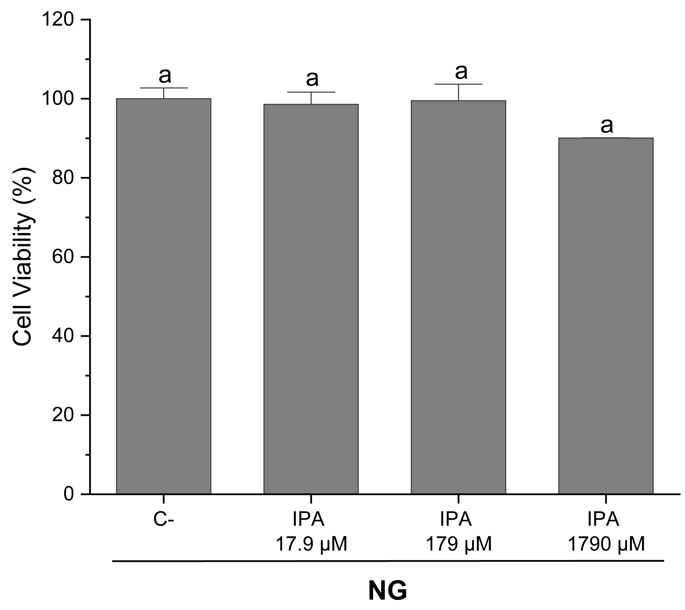 | (B) VP  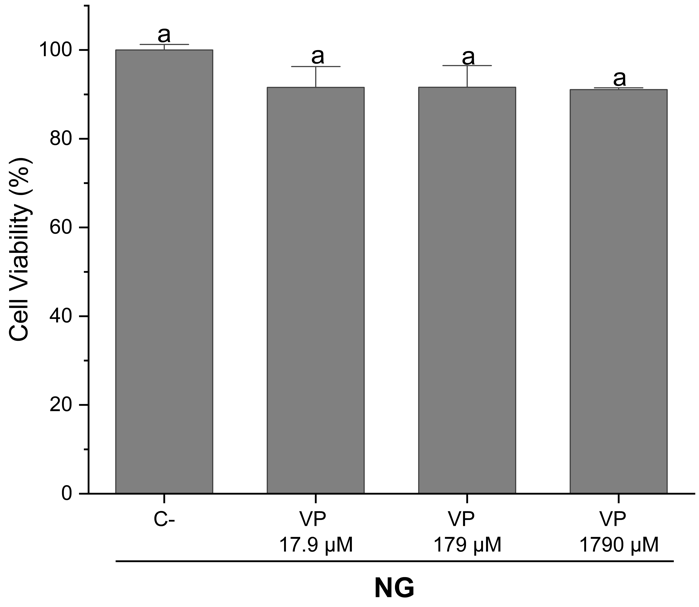 | (C) PM  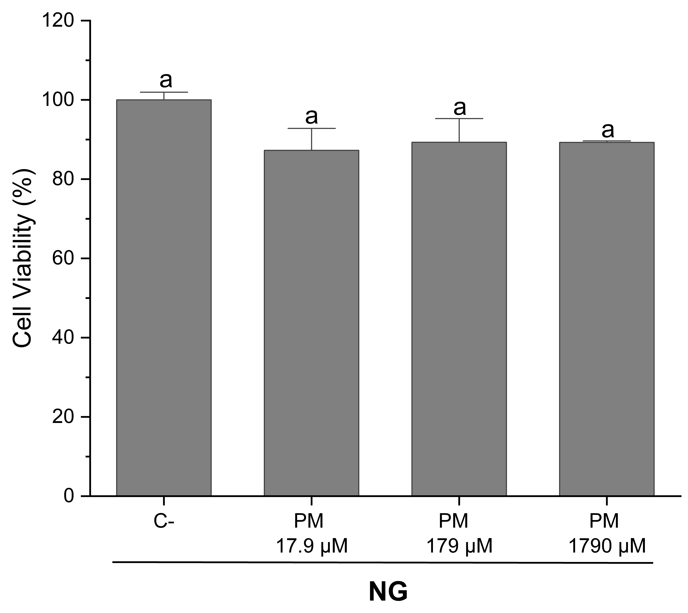 | (D) LLS  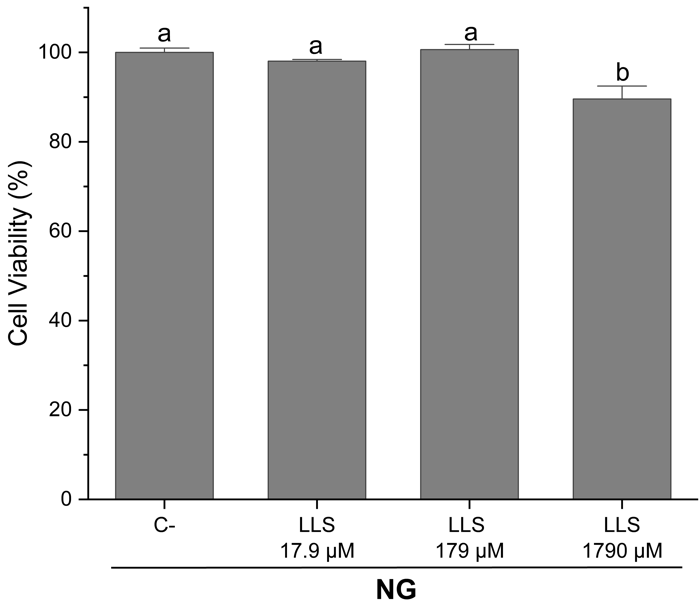 | (E) FNE  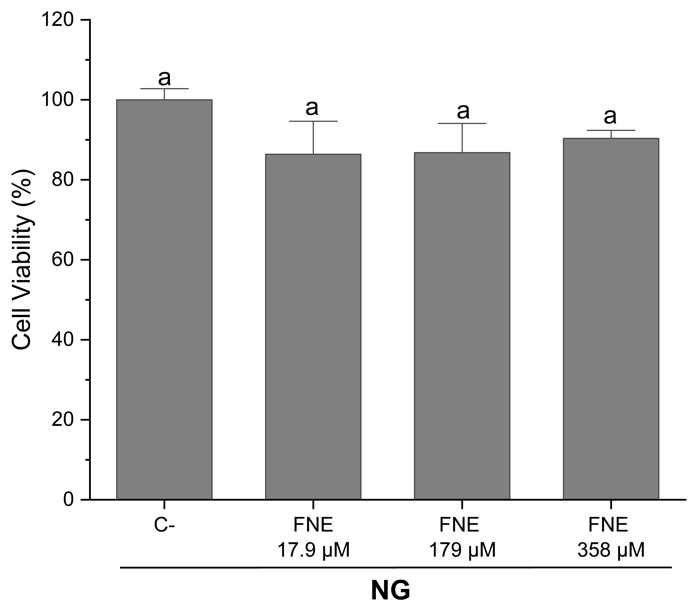 |
| --- | --- | --- | --- | --- |

**Supplementary Figure 2.** Cell viability of HepG2 cells in response to treatments with adzuki bean β-vignin peptides (PM, FNE, VP) and soybean β-conglycinin peptides (IPA, LLS, VP).  HepG2 cell viability for the peptide dosages tested in the dose-response experiment. Results are presented as mean ± standard error of ≥ 3 independent experiments. Different letters indicate means that are significantly different (p < 0.05). C-, untreated control; IPA, peptide Ile-Pro-Ala; VP, peptide Val-Pro; PM, peptide Pro-Met; LLS, peptide Leu-Leu-Ser; FNE, peptide Phe-Asn-Glu.
